# Investigating Cerebellar Modulation of Premovement Beta-Band Activity during Motor Adaptation †

**DOI:** 10.3390/brainsci13111523

**Published:** 2023-10-28

**Authors:** Lynea B. Kaethler, Katlyn E. Brown, Sean K. Meehan, W. Richard Staines

**Affiliations:** Department of Kinesiology and Health Sciences, University of Waterloo, 200 University Ave. W., Waterloo, ON N2L 3G1, Canada; lkaethler@uwaterloo.ca (L.B.K.); kate.brown@uwaterloo.ca (K.E.B.); skmeehan@uwaterloo.ca (S.K.M.)

**Keywords:** cerebellum, motor cortex, premotor, PMd, adaptive plasticity

## Abstract

Enhancing cerebellar activity influences motor cortical activity and contributes to motor adaptation, though it is unclear which neurophysiological mechanisms contributing to adaptation are influenced by the cerebellum. Pre-movement beta event-related desynchronization (β-ERD), which reflects a release of inhibitory control in the premotor cortex during movement planning, is one mechanism that may be modulated by the cerebellum through cerebellar-premotor connections. We hypothesized that enhancing cerebellar activity with intermittent theta burst stimulation (iTBS) would improve adaptation rates and increase β-ERD during motor adaptation. Thirty-four participants were randomly assigned to an active (A-iTBS) or sham cerebellar iTBS (S-iTBS) group. Participants performed a visuomotor task, using a joystick to move a cursor to targets, prior to receiving A-iTBS or S-iTBS, following which they completed training with a 45° rotation to the cursor movement. Behavioural adaptation was assessed using the angular error of the cursor path relative to the ideal trajectory. The results showed a greater adaptation rate following A-iTBS and an increase in β-ERD, specific to the high β range (20–30 Hz) during motor planning, compared to S-iTBS, indicative of cerebellar modulation of the motor cortical inhibitory control network. The enhanced release of inhibitory activity persisted throughout training, which suggests that the cerebellar influence over the premotor cortex extends beyond adaptation to other stages of motor learning. The results from this study further understanding of cerebellum-motor connections as they relate to acquiring motor skills and may inform future skill training and rehabilitation protocols.

## 1. Introduction

The ability to learn and adapt movement is a critical ability, enabling us to successfully interact with the dynamic environments we live in. Motor adaptation uses sensory feedback to determine errors in movement and update the motor plan accordingly to improve or regain the expected movement outcome. Motor cortical areas, including the premotor cortex (PMC) and the primary motor cortex (M1), are responsible for planning and executing movements, respectively; however, regions that are not part of the motor cortex, such as the cerebellum (CB), are crucial in detecting and calculating movement-related errors [1]. 

The dorsal premotor cortex (PMd) is implicated in motor adaptation owing to its role in planning movement parameters such as trajectory, speed, distance, manoeuvres to avoid obstacles, and the grasping actions for movements of varying complexity [2,3,4,5]. Several movement plans can be encoded simultaneously in the PMd, and the PMd is also responsible for selecting and generating the appropriate movement plan [2]. 

The CB has a terminally inhibitory influence over the motor cortex. Excitatory outputs from the dentate nuclei of the CB ultimately influence the activity of inhibitory interneurons in the motor cortex, where increasing the output from the dentate nuclei leads to an overall increased inhibition of the motor cortical output activity. During movement planning, the CB inhibitory control over the PMd [6] and M1 [7] is released. When exposed to a perturbation, the magnitude of the inhibitory release is enhanced during early adaptation, when error signalling is presumably greatest, and declines as error magnitudes decrease with experience. Conversely, the PMd-CB connections have a terminally excitatory influence over the CB and are enhanced during adaptation. In addition, greater attenuation of the predominant CB-PMd inhibition is correlated with faster adaptation to a visuomotor mapping task [6]. 

The theoretical framework for planning and adapting movement assumes that movement plans are based on previous experience and require the integration of multimodal sensory feedback for adaptation to occur [8]. The planned motor command and current sensory state are combined with a forward model to determine the sensations associated with the expected movement outcome [8,9,10]. The forward model result is used to generate a sensory prediction error (SPE) by comparing the predicted sensations against the actual sensations generated by the movement. During task adaptation, the SPE is used to update the forward model, leading to better motor plans and improved movement accuracy given the current state [11,12]. Cerebellar activity during adaptation is related to error detection and processing, specifically using the SPE to inform movement during adaptation [7,13] and updating sensory perceptual models [1].

Visuomotor adaptation tasks are commonly used to measure motor adaptation to a perturbation using the visual feedback of task performance to assess how error feedback is used for the updating of planned motor commands. In such experiments, the subjects train on a task in which a visual or mechanical perturbation disrupts the natural or expected movement outcome, causing significant movement errors. As subjects use sensory feedback to adapt their movements, on a trial-by-trial basis, the errors are reduced until the movement can be consistently and accurately executed [14]. Visuomotor rotation tasks use altered visual feedback and, therefore, require a greater dependence on the visual system feedback. Visuomotor rotation tasks require participants to move a cursor toward a target on a screen, and visual feedback is rotated from the expected trajectory path, typically by 30–60°. Visuomotor rotation tasks encourage greater dependence on the implicit learning processes [15] and can be used to probe activity in the connections between the CB and motor cortical regions, which play a vital role in the implicit learning process [6,16].

Intermittent theta burst stimulation (iTBS) is a technique used to upregulate activity in small clusters of cortical neurons by stimulating the neuronal populations in a pattern that induces temporary plasticity-like effects in the stimulated region. iTBS has been successfully used as an experimental tool to investigate the behavioural and neurophysiological effects of upregulating activity in cerebral and cerebellar cortical regions. Previous research has used iTBS to upregulate activity in the CB and found that doing so increased TMS-induced cortical activity in M1 and improved the rate of adaptation to a novel perturbation on a visuomotor rotation task [17]. 

Beta event-related desynchronization (ß-ERD) refers to the reduction in cortical oscillations in the β band (~13–30 Hz) in response to a particular event. The β band can further be subdivided into low- (~13–20 Hz) and high- (~20–30 Hz) frequency ranges. Evidence suggests lower β frequencies are prevalent primarily in deeper laminar structures and in the basal ganglia (BG) [18,19]. Greater power in low β frequencies, in the BG–cortical loop, is thought to be “anti-kinetic” [20], in keeping with the theory that ß band activity maintains a steady state [21] in the motor cortex, which prevents sporadic activity from causing unplanned movement. In contrast, higher β frequencies are thought to be involved in attentional processes, such as the top–down control of visuomotor processing and anticipation of task-related cues, and greater power in the high β frequencies is associated with maintenance of ‘ready’ posture and faster reaction times [19,22,23]. 

Measuring physiological changes in sensorimotor regions may provide an index of early adaptation and subsequent behavioural changes. However, the relationship between physiological and behavioural changes and how regions external to the sensorimotor cortex can contribute to these physiological adaptive processes is unclear. This study investigated how upregulating activity in the CB affects activity changes in motor planning regions to promote more rapid motor adaptation. Numerous prior studies measured pre- and post-movement power changes across the entire β frequency range (~13–30 Hz) [24,25,26,27,28]; however, given the diverse nature of the role of β frequency bands, we considered both the entire range of β frequencies as well as subdividing the β band to elucidate whether there is a differential impact on each of the high-(β_H_) and low-(β_L_) β frequency ranges. We hypothesized that with CB-iTBS, there would be an enhancement in β_H_- and β-ERD, specifically in the early stage of adaptation, immediately following the introduction of the novel perturbation, compared to no CB-iTBS. 

## 2. Materials and Methods

### 2.1. Participants

Thirty-four participants (fifteen male, nineteen female) were recruited from the University of Waterloo community. Eligible participants were between the ages of 18 and 40, free of neurological pathologies, not taking psychotropic medications, had no history of severe head injury, and had 20/20 or corrected to 20/20 vision. Study procedures were approved by the Research Ethics Board at the University of Waterloo, and participants provided informed written consent before beginning the study.

### 2.2. Experimental Design and Procedures

**Figure 1:**
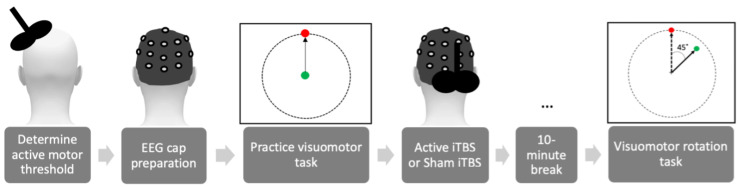
Order of procedures. An example target location is represented by the red circle and the cursor by the green circle.

This study followed a between-groups design, with groups of equal sizes (*n* = 17 per group). Participants were pseudo-randomly assigned to receive either active iTBS (A-iTBS) or sham iTBS (S-iTBS). Participants who had experienced TMS in the past were placed in the A-iTBS group (8 females, average age ± standard error: 22.5 ± 2.1 years old, 2 left-handed). All the S-iTBS participants (11 females, 21.6 ± 2.7 years old, 4 left-handed) were naïve to TMS. Both groups were under the impression they received A-iTBS for the duration of the study. The order of study procedures is depicted in Figure 1. 

Prior to administering iTBS, each participant completed 2 practice blocks of the visuomotor task, with no rotational perturbation. This allowed participants to understand the task timing and the relationship between the joystick and cursor movement and provided a baseline measure for each participant’s unperturbed movement-related β activity and joystick control error. 

### 2.3. Visuomotor Task

**Figure 2:**
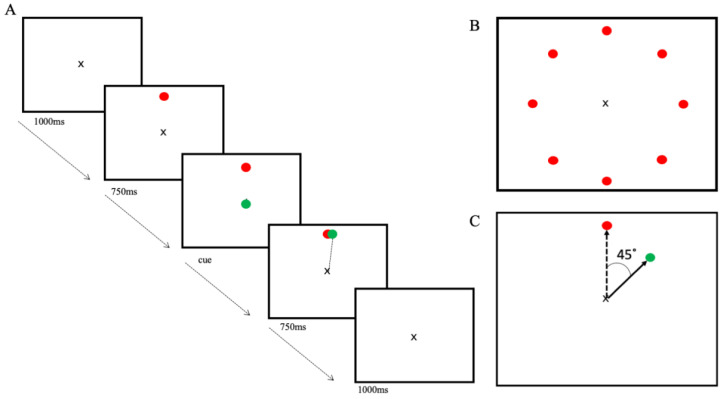
Trial sequence and timing of events. (**A**). The events and timing of an individual trial: 1000 ms of rest, followed by a target (red circle) appearance for 750 ms to prepare for movement, then the cursor (green circle) appearance as the cue to move with 750 ms to allow for execution, followed by 1000 ms of rest for a total of 2000 ms rest between the movement and the appearance of the next target. (**B**). All possible target locations. (**C**). Cursor movement rotation presented in the motor adaptation task.

Participants were seated 70 cm away from the computer screen with the joystick, which was fixed to the table, in their dominant hand and their arm in a neutral position. Using a custom-made program written in LabVIEW (National Instruments, Austin, TX, USA), an ‘x’ was presented in the centre of the computer screen, which represented the starting position for the cursor, a green circle (6 mm). Each trial started with the centre x visible. The target, a red circle (6 mm), would appear on the screen at 1 of 8 locations equidistant from the centre cross and 45° apart (Figure 2B), followed, 750 ms later, by the appearance of the cursor on top of the centre x, which was the cue to move. Targets appeared pseudo-randomly at each position every 8 trials. Participants were instructed to use the joystick to move the cursor through the target, in a straight line, as quickly and accurately as possible, making a striking motion. It was also emphasized to participants to wait until the cursor appeared before moving the joystick. The cursor and target simultaneously disappeared when the cursor passed the outer boundary of the target or when the time limit of 750 ms was exceeded, which concluded the trial. Between each trial, there was a 2000 ms pause, where only the centre x was visible on the screen (Figure 2A).

Each block was 40 trials in length. When participants began training on the visuomotor task, following practice blocks and A-/S-iTBS, they were informed they may notice a difference in the relationship between the way they moved the joystick and the cursor movement on the screen, but the goal and instructions remained the same. Ten blocks of the training task were completed for a total of 400 trials. Brief breaks between blocks were given as needed. 

### 2.4. iTBS

The MagPro R30 stimulator (MagVenture, Alpharetta, GA, USA), with a 70 mm figure-eight coil, was used to deliver iTBS to the posterior lobule in the CB, 1 cm inferior and 3 cm lateral from the inion on the ipsilateral-dominant-hand side (CB-iTBS). For A-iTBS, the stimulus intensity was set at 80% of the participant’s active motor threshold (AMT). AMT was determined as the intensity that evokes a motor-evoked potential (MEP) of at least 200 μV in five out of ten consecutive trials during a ~10% muscle contraction in the dominant hand first dorsal interosseous muscle (FDI) when applying TMS to the location within the motor cortex that elicits the most reliable twitch in the FDI (M1FDI). The stimulus intensity of the coil for S-iTBS was set at 20% of the participant’s AMT and placed perpendicular to the skull at the same location used for A-iTBS [17,29]. Motor threshold was determined without the EEG cap since the placement of the coil over the posterior lateral CB was not affected by the cap.

### 2.5. Data Acquisition

The EEG data were recorded from a 32-channel electrode cap (Quik-Cap, Neuroscan, Compumedics, Charlotte, NC, USA) according to the International 10–20 system. Specifically, ten recording electrodes located over the sensorimotor regions (FCZ, FC3, FC4, CPZ, CP3, CP4, C3, CZ, C4) and FP1, which was used to identify blinks and facial movement, were referenced to electrodes placed on the right and left mastoids. Impedances were maintained below 5 kΩ, and continuous EEG data were collected, filtered (DC-200 Hz, 6 dB octave roll-off), and digitized at 1000 Hz (SynAmps2, Scan 4.5, Compumedics Neuroscan, Charlotte, NC, USA) before being stored off-line for analysis. 

Behavioural data were measured as voltage changes from the joystick movement, representing the direction/distance moved and recorded in the LabVIEW program used to run the visuomotor task. The cursor positional data were collected at approximately 90 Hz.

### 2.6. Data Analysis

Mistrials where the participant either moved before being cued to do so or did not move at all within the 750 ms movement time window were removed from both the behavioural and EEG analysis. 

### 2.7. Behavioural Analysis

The angular error (AE) was measured at the peak velocity of the movement as the angular difference between the cursor location and the ideal trajectory between the cursor start location and target location. Response time (RT) was calculated as the difference in time between the appearance of the cue signifying to move and the onset of movement, the time at which the cursor left the initial starting point.

Using angular error, the adaptation rate was determined with an asymptotic regression function:

*y*(*x*)~*y*_*f*_ + (*y*_0_ − *y*_*f*_)*e*^−exp(logα)*x*^

where *x* is the error, *y*_*f*_ is the AE asymptote, *y*_0_ is the starting AE, and logα is the rate of AE decay. 

An average of 15% of the trials were flagged as mistrials and omitted for each participant, and an average of 3% of the trials were omitted as outliers (greater than 2 standard deviations from the mean of each bin of 8 trials). 

Using the final block (40 trials) of practice with no rotation, the first block of training with the rotation, and the final block of training with rotation, β-ERD was analysed during the movement planning phase (P1) (following the appearance of the target but before the cue to move) and in the movement preparation/pre-movement phase (P2) (500 ms preceding the onset of movement). For both time windows, β power was evaluated across the entire β range as well as separated into β_H_ and β_L_ frequency ranges. 

### 2.8. EEG Analysis

Data were imported into EEGLAB 2021.0 [30], run on MATLAB Simulink, and band-pass filtered between 1 and 50 Hz. An independent component analysis (ICA) was run on the continuous EEG dataset for each individual to identify the component containing blinks, and after manual inspection and confirmation, the component was removed. After filtering and ICA blink removal, all datasets were manually inspected, and portions of the data that were corrupted by muscle activity or other spontaneous noise were removed. Epochs were generated by splicing the data 1750 ms prior to each cue to move and 1750 ms following. 

**Figure 3:**
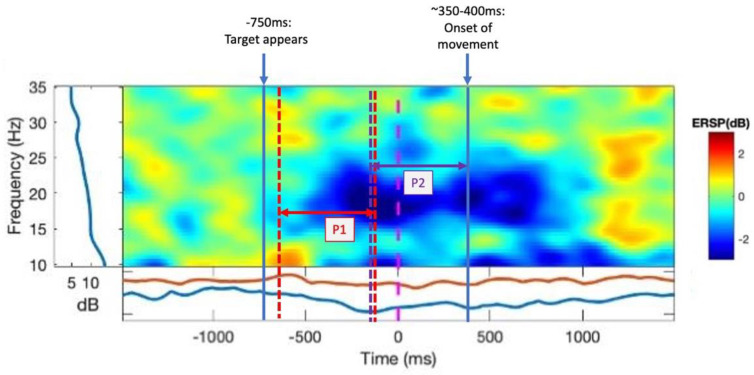
Individual ERSP plot. Example ERSP from an individual participant during practice (no rotation). The frequency is shown on the y-axis and time (ms) on the x-axis. The target appears at −750 ms, and the cue to move is at 0 ms. The baseline period is the time prior to the target. The index for the colour map is shown to the right of the graph. Lower power (dB) indicates greater ERD.

A power analysis was carried out using an event-related spectral perturbation (ERSP) analysis. We analysed the β power in the FC3 electrode (or FC4 electrode for left-hand-dominant participants), of which the most dominant source contributing is the PMC. ERD in the β band (13–30 Hz) in the time window of interest was measured as a change in power from the baseline period, which was the rest period of each trial or the first 1000 ms of the epoch, the interstimulus interval prior to the presentation of the target (Figure 2A, first panel). We measured ERD in the β range during P1 of a trial: from −630 ms to −100 ms, respective to the cue to move, capturing the participant’s awareness of the appearance of the target location up until the cue to move, representing movement planning. β-ERD was also measured during P2 (−500 ms–0 ms with respect to the onset of movement), representing movement preparation (Figure 3). To assess how introducing the perturbation and iTBS affected β-ERD, we measured the change in ERD from the last block of practice (no visual feedback rotation) to the first block of training (with rotation of visual feedback) as our measure of ERD change in early training (early) to represent the initial adaptation to the perturbation. The change in ERD from the first block of practice to the last block of training was our measure of ERD change in late training (late) once adaptation has occurred and participants are more familiar with the task. As different sections of the beta band may represent distinct neural processes [19], we conducted a second set of analyses that separated the beta range (13–30 Hz) into a lower beta range, spanning 13–20 Hz (β_L_-ERD), and a higher range, from 20 to 30 Hz (β_H_-ERD).

### 2.9. Statistical Analysis

Shapiro–Wilk’s test and Levene’s test were run prior to statistical testing to confirm that all datasets met the assumption of normality and homoscedasticity, respectively. A one-tailed independent-samples *t*-test was run to determine whether the A-iTBS group was able to adapt to the perturbation faster than the S-iTBS group. Group was treated as the independent variable and adaptation rate was the dependent variable. A 2-way mixed-model ANOVA was run to assess the effect of the group and block on response times. Group was treated as the between-subjects factor and Block as the within-subjects factor, with 3 levels: pre, early, late. The Greenhouse–Geisser epsilon correction was applied to correct for violations of sphericity between blocks. In addition, 2-way mixed-model ANOVA tests were run to determine whether CB-iTBS influenced the change in ß-ERD during P1 and P2 during early and late training. Training was treated as the within-subjects factor with 2 levels (early/late), and group was treated as the between-subjects factor with 2 levels (A-iTBS/S-iTBS).

## 3. Results

### 3.1. Rate of Adaptation/Response Time

**Figure 4:**
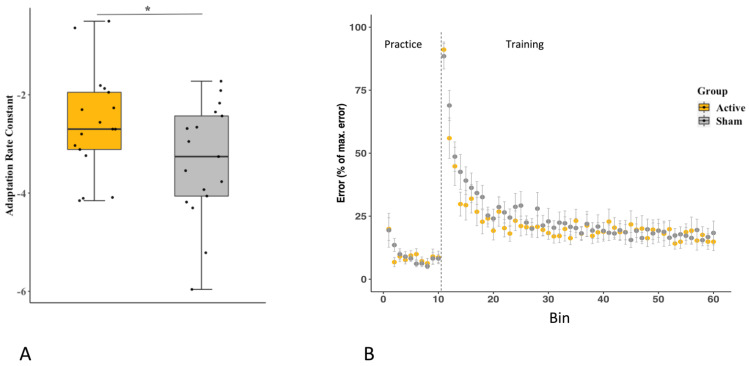
(**A**). Adaptation rate between groups. Boxplot of error decay rate, used as the measure of adaptation rate, in each group. Datapoints on the boxplots each represent an individual participant’s adaptation rate constant. * indicates significant difference *p* < 0.05 (**B**). Error across practice and training. Group error rates plotted over time. Each data point represents the group average median error in each bin. Error values are normalized to the maximum median error within each participant for ease of viewing the comparison between groups. Error bars show the standard error of the mean. Bins 1 through 10 represent the pre-perturbation/practice period, bins 11 through 60 show the training period.

An independent-samples *t*-test revealed a significantly greater rate of adaptation in the A-iTBS group compared to the S-iTBS group (t_32_ = 2.04, *p* = 0.025, A-iTBS mean = −2.58, SE = 0.26, S-iTBS mean = −3.22, SE 0.34, Cohen’s D = 0.7) (Figure 4). The ANOVA for response time revealed no significant effect of Group (F_1,32_ = 1.85, *p* = 0.18, η^2^ = 0.03) or interaction between Group and Block (F_1.86,59.43_ = 0.50, *p* = 0.59, η^2^ = 0.007), but there was a significant main effect of Block (F_1.86,59.43_ = 6.8, *p* = 0.003, η^2^ = 0.08). Tukey’s HSD revealed that the response time was shorter in the late relative to early training period (*p* = 0.02, early = 373 ± 18.6, late = 308 ± 15.8 ms).

### 3.2. β-ERD—Movement Planning Prior to Cue Onset

**Figure 5:**
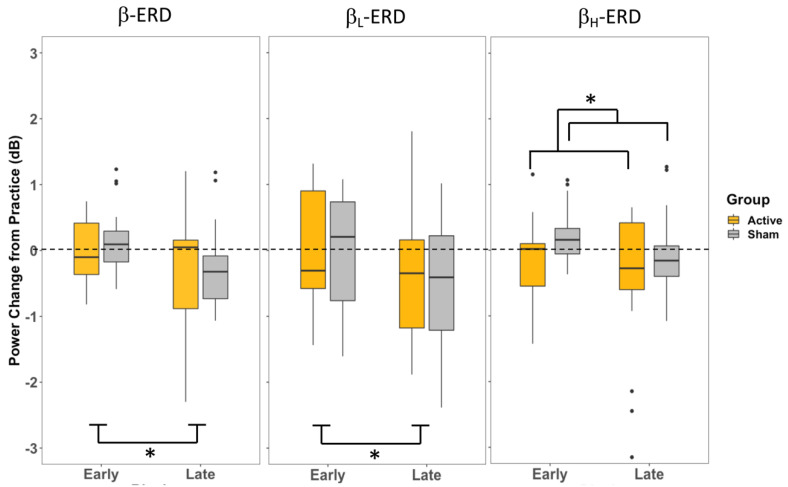
Change in β-ERD at P1 for each the entire β band and separated β_L_ and β_H_ at beginning of visuomotor task training (early) and the end of training (late), measured from the FC3 electrode. * indicates a significance of *p* < 0.05.

The two-way mixed model ANOVA (Group × Training) for β-ERD in P1 revealed a significant main effect of Training (early/late) (F_1,32_ = 6.57, *p* = 0.015, η^2^ = 0.078), where the late training period had a greater decrease in power than the early training period (Figure 5). There was no significant effect of Group (A-iTBS/S-iTBS) (F_1,32_ = 0.619, *p* = 0.44, η^2^ = 0.011), and the interaction (Group × Training) was not significant (F_1,32_ = 0.043, *p* = 0.84, η^2^ = 0.0005). The ANOVA (Group × Training) for β_H_-ERD in P1 similarly revealed no significant interaction (F_1,32_ = 0.031, *p* = 0.86, η^2^ = 0.0005). There was not a significant main effect of Training (F_1,32_ = 2.768, *p* = 0.106, η^2^ = 0.045), but there was a significant effect of Group (F_1,32_ = 5.703, *p* = 0.023, η^2^ = 0.075), where the A-iTBS group had a greater decrease in power than the S-iTBS group (Figure 5). The ANOVA (Group × Training) for β_L_-ERD in P1 revealed no significant interaction (F_1,32_ = 0.055, *p* = 0.82 η^2^ = 0.0004). There was a significant effect of Training (F_1,32_ = 8.916, *p* = 0.005, η^2^ = 0.064), where the late training period had a greater decrease in power than the early training period, but no effect of Group was found (F_1,32_ = 0.130, *p* = 0.721, η^2^ = 0.003) (Figure 5).

### 3.3. β-ERD—Movement Preparation Relative to Cue to Move

**Figure 6:**
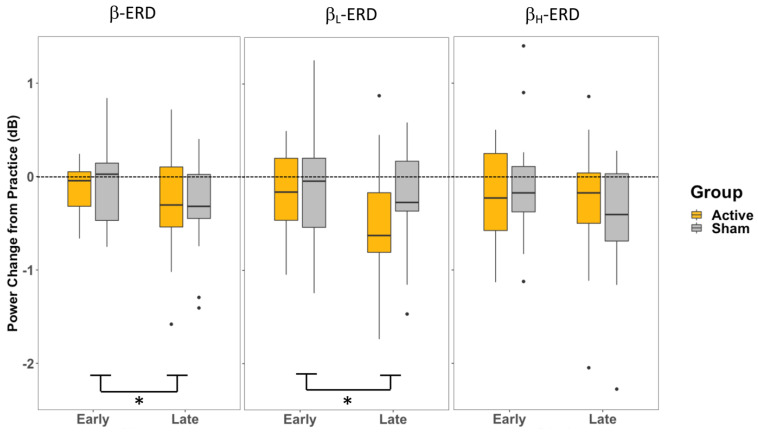
Change in β-ERD at P2 for each the entire β band and separated β_L_ and β_H_ at beginning of visuomotor task training (early) and the end of training (late), measured from the FC3 electrode. * indicates a significance of *p* < 0.05.

The two-way mixed model ANOVA (Group × Training) for β-ERD in P2 revealed a significant main effect of Training (early/late) (F_1,32_ = 6.96, *p* = 0.013, η^2^ = 0.066), where the late training period had a greater decrease in power than the early training period (Figure 6). There was no main effect of Group (A-iTBS/S-iTBS) (F_1,32_ = 0.173, *p* = 0.68, η^2^ = 0.004), and the interaction (Group × Training) was not significant (F_1,32_ = 0.224, *p* = 0.64, η^2^ = 0.002). The ANOVA (Group × Training) for β_L_-ERD in P2 similarly revealed a significant main effect of Training (F_1,32_ = 7.183, *p* = 0.012, η^2^ = 0.058), where the late training period had a greater decrease in power than the early training period, and no effect of Group (F_1,32_ = 0.515, *p* = 0.478, η^2^ = 0.012) nor an interaction between these (F_1,32_ = 0.602, *p* = 0.44, η^2^ = 0.005) (Figure 6) were found. The ANOVA (Group × Training) for β_H_-ERD in P2 similarly revealed no significant main effects of Training (F_1,32_ = 2.175, *p* = 0.15, η^2^ = 0.024) or Group (F_1,32_ = 0.065, *p* = 0.80, η^2^ = 0.001), nor an interaction between these (F_1,32_ = 1.109, *p* = 0.30, η^2^ = 0.012). 

## 4. Discussion

The general results from this study are consistent with previous literature identifying the role of the CB in motor adaptation [17,31] and showing that enhancing cerebellar activity with iTBS improves the rate of adaptation on a visuomotor rotation task [17]. This improved rate of behavioural adaptation following CB-iTBS was accompanied by an increase in β_H_-ERD, whereas motor adaptation generally was associated with an increase in β- and β_L_-ERD. This study adds to the literature by contributing to our understanding of how upregulating activity in the CB (directly or indirectly) affects activity changes in the motor planning areas, namely the PMC, during motor preparation. 

Although there was a significant difference in adaptation rate between the groups, the rate in both groups was notably quicker, and the difference between the A-iTBS and S-iTBS adaptation rates was less pronounced than in the study by Koch et al. [17]. This may be due to differences in task difficulty. The current study used a handheld rather than a finger-controlled joystick, which was likely easier to control. In addition, the 45° rotation and target separation used here may have been easier to adapt to than the 30° rotation and 60° target separation used by Koch et al. [17]. The handheld joystick used for the current study was selected to increase the real-world relevance of the task, and most studies measuring β-ERD or other motor planning and preparation activity measures used tasks requiring wrist and forearm flexion/extension. The 45° rotation with targets placed 45° apart may have helped participants visualize the rotation and how to correct for it since they could imagine where the neighbouring target would be located and move as though they were aiming for that location. If the rotation was seemingly more arbitrary, that strategy would not have been possible and may have increased the task difficulty, which might have resulted in a greater between-group difference in adaptation rate. The task in the current study may have been easy enough that the CB-iTBS was less beneficial than it would have been in a more challenging task. Given greater task difficulty, the adaptation rate would have been shallower, and therefore, a greater difference between groups may have been observed. 

These behavioural effects were accompanied by electrophysiological changes reflected in the desynchronization of β band activity recorded over premotor cortical sites. In both the movement planning prior to the cue (P1) and the movement preparation following the cursor presentation (P2), the overall β power (13–30 Hz) and β_L_ (13–20 Hz) power were only influenced by the stage of training, with less of a change in ERD in the early stages of training when adapting to the perturbation and a greater increase in ERD during the late training period (Figure 5 and Figure 6). These effects were not influenced by the CB-iTBS. In contrast, during movement planning prior to the cue to move (P1), β_H_ (20–30 Hz) power was influenced by the CB-iTBS and did not change with training; it was elevated in both the early and late training stages following the CB-iTBS, although there was no significant effect observed in either training or group in the β_H_ in P2. The observed changes in β-ERD provide insights into the relationship between the stages of training, the effects of CB-iTBS, and cortical activity in each of the movement planning and movement preparation activities during motor adaptation.

### 4.1. P1—Movement Planning Prior to Cue Onset

The increase in late-training β-ERD measures, which are exhibited as a decrease in power relative to that in practice, may reflect an increase in motor cortical excitability, which can occur through LTP-like effects from 20 min of motor training [32,33]. Decreased synchronized activity in the PMC could also result from an overall enhancement of network excitability in the CB-PMd network. This explanation suggests that the enhancement of the CB-PMd network activity is not specific to adaptation but movement planning in general, so the enhanced CB activity may have enhanced PMC activity for the duration of the effects of the iTBS. iTBS has been shown to increase excitability in the motor cortex for approximately 60 min following stimulation [34]. 

The only effect of Group observed in the β-ERD analysis was seen during motor planning prior to the cue to move (P1) β_H_-ERD, where A-iTBS showed a greater amount of β_H_-ERD than S-iTBS across both the early and late stages of training. β_H_ is thought to be involved in attentional processes, such as the top–down control of visuomotor processing and the anticipation of task-related cues. Greater β power in the β_H_ frequencies is associated with the maintenance of ‘ready’ posture and faster reaction times [19,22,23]. CB-iTBS affected this top–down, attentional control process, as seen by the A-iTBS group increase in the release of β-ERD. However, given the lasting effects of iTBS, this enhanced β_H_-ERD likely persisted as long as the effects of iTBS lasted in the CB. This effect is seen during motor planning (P1) in β_H_, where there is a difference between the A-iTBS and S-iTBS groups across both the early and late training stages (Figure 5). During the movement planning stage, represented by P1, participants are aware of the target location and can start to plan their movement while still attentively awaiting the cue to move. This actively involves the attentional and anticipatory processes reflected in β_H_. 

Variability across participants in each β and β_H_ (Figure 5) may be explained by individual differences in attention, motivation, or strategy. This is also supported by the presence of anticipatory neurons in the PMd [35,36], a region that generates β_H_-band activity and substantially contributes to the β measures at the FC3 electrode. Participants who were motivated and awaiting the target appearance with eager anticipation may have exhibited a greater β_H_-ERD, an overall increase in power generated by these anticipatory neuron populations. However, participants who were less motivated to move promptly following the cue would have less contribution from the anticipatory neuronal populations to the β_H_-ERD. 

Low β is more prevalent in the deeper laminar structures and is notably present in the BG [18,19]. Greater power in β_L_, in the BG–cortical loop, is thought to be “anti-kinetic” [20], in keeping with the theory that β-band activity maintains a steady state [21] in the motor cortex, which prevents sporadic activity from causing unplanned movement. The greatest contributor to β_L_ measured at the FC3 electrode is the activity in the BG–cortical loop, which modulates the premotor cortical inhibitory control during motor planning. Decreases in β_L_ power in the late stages of training, regardless of the group (Figure 5), may reflect some release of steady-state inhibitory control resulting from the frequent, successive movement generation. 

### 4.2. P2—Movement Preparation after Cue to Move

While a change in β-ERD and β_L_-ERD across time was observed, the β_H_-ERD during the later stages of movement preparation (represented by P2: −500–0 ms relative to movement onset) stays unperturbed by either the CB-iTBS or the familiarity of the perturbation to the visuomotor task (Figure 6). This constancy in β_H_ suggests the processes contributing to β_H_ during movement preparation and generation of the motor command are less affected by the novelty or familiarity of a perturbation. This could also suggest that the task still required elevated attention, even though participants were able to consistently and accurately perform the visuomotor task. These later stages of learning may still be modulated by the processes contributing to β_H,_ similar to the early stages of learning. Alternatively, it is also possible that the β_H_-contributing processes are less involved at a measurable level during the later stages of movement preparation compared to the earlier movement planning phase, where changes in β_H_ were observed. 

Changes in β and β_L_ during motor preparation (P2) at late training compared to early training were observed (Figure 6); however, they did not appear to be mediated by the increase in cerebellar activity, given the similar trend of an increase in ERD in both groups. The overall effect of training in movement preparation (P2) in β and β_L_ is likely driven by the change in steady-state control throughout the movement planning and preparation periods. Similar to how changes in movement planning (P1) were observed across the training period, the same effect of some release of steady-state control due to frequent movements affects movement preparation (P2) as well. Since P2 represents the late stage of movement preparation and the transition into movement execution, it is probable that the release of inhibitory control lasts throughout the movement planning period and the generation of movement. 

Although sensory processing is affected by the magnitude of error [27], it may be that model updating and the planning of the subsequent movement result in an alteration in the pattern of inhibitory control in the preparatory network but not a change in the overall magnitude of inhibitory control or the release of inhibitory control. In other words, the microcircuit’s activity may change, and the inhibitory activity may migrate, but the overall amount of inhibitory control stays the same. It has been proposed that the sensory integration and model updating/planning circuits function separately from one another [27], so the sensory processing may be affected by the changes in error during adaptation, but the changes in one circuit may not be linearly reflected by changes in the other. 

There are many other factors that could be contributing to both the β-ERD and the rate of adaptation. There are distinct neural networks and patterns of activity specific to implicit and explicit learning processes, which rely on differing contributions, notably from the CB, dlPFC, and distinct regions of the basal ganglia [37,38]. Although the study was designed to target primarily implicit learning, participants presumably employed both processes, but with varying reliance on one or the other. The strategy and resulting distinct patterns of activity in these other brain regions may differently affect the inhibitory activity in the PMC or contribute to adaptation in alternate ways. 

### 4.3. Limitations

For analysis purposes, we normalized the β power during the planning periods to the β power during the practice (no rotation) to observe whether iTBS had an impact on power changes during adaptation. Prior to normalizing the data, there were group differences in β power when measured across all timepoints: practice, early, and late training. When comparing the practice levels of β, the groups were not significantly different from one another in β or β_H_; however, β_L_ was statistically different between the A-iTBS and S-iTBS groups in the movement planning (P1) phase (t_32_ = −2.73, *p* = 0.03, A-iTBS = −1.39 ± 0.28, S-iTBS = −0.558 ± 0.25). These group differences, which existed prior to training, may have contributed to the rate of adaptation. A greater power reduction in β activity during movement planning might indicate that an individual is better primed to adapt more rapidly to a perturbation, resulting from a more substantial network connectivity and activity in the CB–cortical or cortical–cortical networks, which contribute to adaptation. A final potential limitation to the current work is the possibility that not all participants in the active group responded to the cerebellar iTBS. Therefore, our effect sizes may underestimate the influence of cerebellar modulation over adaptation and β-ERD. Future work could benefit from titrating iTBS responders from non-responders using cerebellar–cortical dual-coil paired-pulse TMS.

## 5. Conclusions

The main findings from this research were confirming that CB-iTBS can improve the rate of adaptation in a visuomotor adaptation task, and the β_H_-ERD during movement planning was affected by the CB-iTBS, which was shown in the significant increase in the A-iTBS group’s β_H_-ERD compared to that in the S-iTBS group. The β- and β_L_-ERD were unaffected by the CB-iTBS but increased during the training period following the rotation of visual feedback, exhibiting a greater ERD later in the training period than early in the training period. We hypothesize that the β_H_-ERD during P1, the time following the target appearance but prior to the cue to move, was influenced by the increase in CB activity. We conclude that CB-iTBS modulated β_H_-generating activity in the PMC to increase the amount of ERD, reflective of a release of inhibitory control. Gaining a better understanding of how the CB and PMC communicate to promote motor adaptation could contribute to identifying the deficits present in neurological disease or damage and to providing targeted rehabilitation to individuals suffering from movement-related injuries.

## Data Availability

The datasets generated for this study are available on request to the corresponding author.
